# Mechanism of Qingchang Suppository on repairing the intestinal mucosal barrier in ulcerative colitis

**DOI:** 10.3389/fphar.2023.1221849

**Published:** 2023-08-22

**Authors:** Jingyi Shan, Suxian Liu, Haoyue Liu, Jianye Yuan, Jiang Lin

**Affiliations:** ^1^ Department of Gastroenterology, Longhua Hospital, Shanghai University of Traditional Chinese Medicine, Shanghai, China; ^2^ Institute of Digestive Diseases, Longhua Hospital, Shanghai University of Traditional Chinese Medicine, Shanghai, China; ^3^ Department of Intensive Care Unit, Longhua Hospital, Shanghai University of Traditional Chinese Medicine, Shanghai, China

**Keywords:** ulcerative colitis, Qingchang Suppository, active ingredients, intestinal mucosal barrier, mechanism

## Abstract

Ulcerative colitis (UC) is a refractory inflammatory bowel disease, and the outcomes of conventional therapies of UC, including 5-aminosalicylic acid, glucocorticoids, immunosuppressants, and biological agents, are not satisfied with patients and physicians with regard to adverse reactions and financial burden. The abnormality of the intestinal mucosal barrier in the pathogenesis of UC was verified. Qingchang Suppository (QCS) is an herbal preparation and is effective in treating ulcerative proctitis. The mechanism of QCS and its active ingredients have not been concluded especially in mucosal healing. This review elucidated the potential mechanism of QCS from the intestinal mucosal barrier perspective to help exploring future QCS research directions.

## 1 Introduction

Ulcerative colitis (UC) is a chronic idiopathic inflammation of the colon and rectum (Eisenstein, 2018; Kobayashi et al., 2020). The World Health Organization (WHO) has recognized UC as one of the refractory diseases (Kobayashi et al., 2020). Several clinical studies demonstrated UC patients who achieve mucosal healing (MH) have significantly lower rates of clinical recurrence, hospitalization, surgery, colonic dysplasia, and tumors (Shah et al., 2016; Kobayashi et al., 2020). The American College of Gastroenterology (ACG) introduced MH as a new target for UC treatment in UC clinical guidelines in 2019 (Ungaro et al., 2019).MH is strongly associated with the intestinal mucosal barrier (Boal Carvalho and Cotter, 2017). Thus, repairing the intestinal mucosa is important for the regression of UC. Qingchang Suppository (QCS) is an herbal formula preparation. It is composed of Indigo Naturalis, purslane; Herba Portulacae, Radix Notoginseng; *Panax notoginseng* (Burk.) F. H. Chen Ex C. Chow, Gallnut; Galla Chinensis and borneol; and Borneolum Syntheticum in a ratio of 2:2:5:5:1 under the guidance of TCM theory (Dai et al., 2010; Xu et al., 2019; Zhou G. et al., 2021).

Based on clinical studies, QCS therapy shows good treatment results in clinical symptoms’ relief, patient satisfaction, and recurrent rate decrease. Clinical symptoms’ relief includes decrease in pain, hemorrhage, and diarrhea, a satisfactory patient outcome (Xu et al., 2019).

According to published experimental research articles (Lu and Xie, 2010; Han et al., 2016; Sun et al., 2018; Yu et al., 2021), QCS plays roles in repairing barrier function, suppressing colonic permeability, ameliorating colonic hypoxia, and decreasing colonic micro-vascular permeability (VP).

Mucoal healing is the goal of UC treatment. It has been reported that QCS could promote the colonic mucosa repair in the clinical trials and the animal experiments. This review tends to systematically elucidate the mechnisms of QCS and its ingredients on repairing the colonic mucosa.

## 2 Damaged intestinal barrier in UC

The intestinal barrier is an important interface between the body and the external environment for preventing the invasion of pathogenic antigens and plays a crucial role in maintaining internal homeostasis (Du et al., 2015). The intestinal physical barrier is composed by four sections: the chemical barrier, the mechanical barrier, the immune barrier, and the microbial barrier (Yao et al., 2021). The integrity of the intestinal epithelium is essential for the intestine to function as a barrier (Salvo Romero et al., 2015), and the destruction of tight junctions (TJs) of intestinal epithelial cells increased the intestinal permeability (Miner-Williams and Moughan, 2016; Xiao et al., 2018). TJs are the most important structure and composed of occludin, claudin, and ZOs proteins (Vancamelbeke and Vermeire, 2017), whose integrity is essential for the functioning of the intestinal mucosal barrier (Otani and Furuse, 2020). Several studies have proven that the disruption of the TJs can cause structural and functional damage of the intestinal mucosal barrier (Fukui, 2016; Li et al., 2020a; Li et al., 2020b).

It is now commonly accepted that the abnormality of the intestinal mucosal barrier is the basic pathogenesis initiating and promoting the development of UC (Ungaro et al., 2017; van der Post et al., 2019). The increased epithelial permeability (EP) can lead to intestinal mucosal barrier dysfunction. Intestinal infections and inflammatory factors can induce abnormal intestinal mucosal barrier function, leading to intestinal mucosal permeability increased, and a large number of bacteria and antigens are transported into the lamina propria, activating abnormal mucosal immune responses (Li et al., 2022), and intestinal mucosal barrier function is damaged (Mankertz and Schulzke, 2007; Al-Sadi et al., 2009).

## 3 Clinical research of QCS and its active ingredients

From 1990 to 2020, 11 controlled clinical studies were conducted (Li and June 2018). Especially, in 2020, active mild-to-moderate UC patients were enrolled in a prospective, randomized, positive drug parallel control trial. The study demonstrated that the efficacy, effective time, and course of clinical remission were not inferior to those of SASP suppository (Dai, 2020).

According to the results of HPLC-MC/MC analysis, QCS contains multiple bioactive compounds including indirubin, notoginsenoside R1, ginsenosides Rb1 and Rg1, and gallic acid α (Sun et al., 2018) (Table 1). Indirubin has a significant anti-inflammatory activity (Gao et al., 2016). Notoginsenoside R1, ginsenosides Rb1 (GRb1) and Rg1 are active ingredients in Radix notoginseng. Notoginsenoside R1 could down-regulate vascular endothelial growth factor (VEGF) and matrix metalloproteinase-2 (MMP-2) and promote the regeneration of endothelial cells (ECs) (Chen et al., 2004). GRb1 and Rg1 could induce the production of NO by motivating the PI3K/Akt/eNOS signal pathway to promote arginine transformation in endothelial cells, so as to increase the amount of vessels in rats (Pan et al., 2012). Our recent research found that *Panax notoginseng* could downregulate VEGFA and inhibit the Rap1GAP/TSP1 signaling pathway to promote EP repairment (Wang S. et al., 2018). Gallic acid (GA), an active ingredient of gallnut, could also ameliorate oxidative stress and inflammation and regulate proliferation and apoptosis of the colonic epithelia (Chang et al., 2015). Purslane possesses a wide range of pharmacological effects, such as antimicrobial, antioxidant, anti-inflammatory, antiulcerogenic, and anticancer activities (Zhou et al., 2015) (Table 2).

**TABLE 1:** Characterization and quantification of the major biochemical components from Qingchang Suppository by LC-ESI-MS/MS.

Peak no.	*tR* (min)	Compound	[M-H]-*m/z*	[M + HCOO] –*m/z*	MS/MS data (measured from [M-H]-)	Content (mg/g) *b*
1	1.67	Gallic acid	169.0		125.2	
2	5.57	Notoginsenoside R1		977.4500	769.6 and 619.6	11.358
3	5.87	Ginsenoside Rg1	845.6		799.5 and 627.6	6.482
4	7.31	Ginsenoside Rb1	1,108.55		459.4 and 203.0	0.002
5	10.69	Indirubin	261.0			0.001

**TABLE 2:** Efficacy of the active ingredients of QCS.

Botanical drug	Active ingredient	Effects
Indigo; Indigo Naturalis	Indirubin	anti-inflammation, enhancing the expressions of claudin-2 and antimicrobials, increasing SCFA level, protecting the intestinal mucosal barrier
Pseudo-ginseng; *Panax notoginseng* (Burk.) F. H. Chen Ex C. Chow	Notoginsenoside R1	Downregulate VEGF and MMP-2; promote IEC regeneration and EP repairment; and protect the intestinal mucosal barrier
	Ginsenosides Rb1 and Rg1	Promote arginine transformation in IEC; antioxidant and antiapoptotic activity; gut microbiota composition; and relieve the immune disorder
Gallnut; Galla Chinensis	Gallic acid	Anti-inflammation, antimutation, antioxidation, and anti-free radical; repair the mucosal barrier; decrease endoplasmic reticulum stress; protect the intestinal mucosal barrier; antibacterial, anti-inflammatory, and antioxidant activity; and upregulate glutathione levels and activities of detoxifying enzymes

## 4 Effect and mechanisms of QCS on repairing the intestinal barrier

### 4.1 Effect of QCS against the increased colonic permeability

The most commonly used method to assess intestinal mucosal barrier function is the measurement of intestinal mucosal permeability with fluorescein-dextran 4,000 (FITC-dextran 4,000, FD-4), D-lactate, or diamine oxidase (DAO). FD-4 is an organic fluorescent pigment commonly used in immunofluorescence and flow cytometry to bind to different antibodies with the help of isothiocyanate reactive groups. D-lactate is a metabolite of many bacteria in the gastrointestinal tract. Mammals lack D-lactate dehydrogenase and cannot metabolize it rapidly, so the amount of D-lactate in the body under normal condition is small and relatively stable. When the intestinal mucosal barrier is disrupted, more D-lactate enters the circulation through the damaged intestinal mucosa and the blood D-lactate level increases significantly.

DAO is an intracellular enzyme in mammalian intestinal mucosal cells. When the mucosal cells are damaged, the intracellular DAO is released into the blood and the serum DAO is increased. The higher serum DAO, the severer mucosal damage as well as higher permeability of the intestinal mucosa (Xun et al., 2015). In addition, it has been reported that the level of endotoxin in circulation is positively associated with the degree of permeability of the intestinal mucosa (Tornai et al., 2017).

**FIGURE 1:**
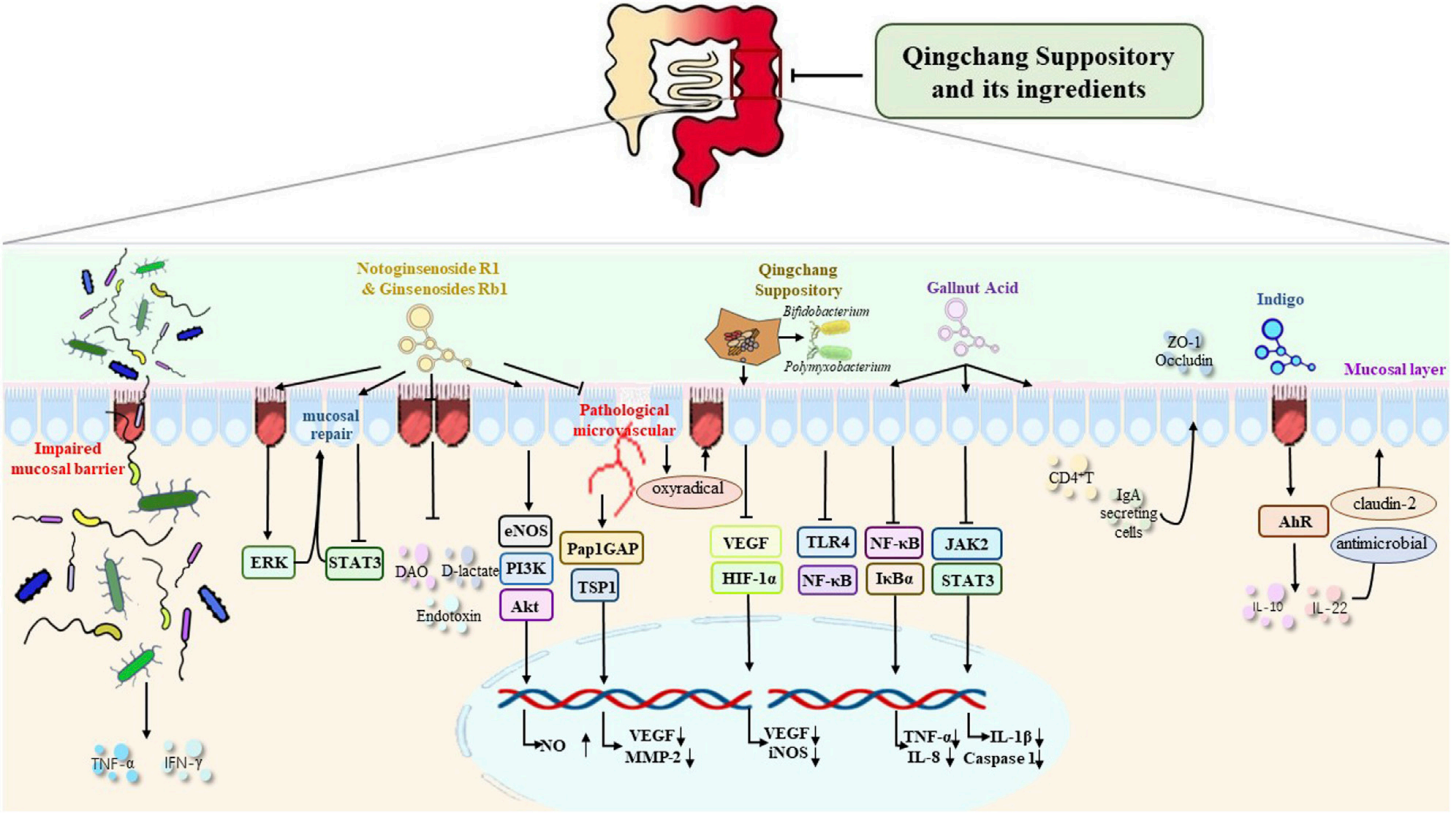
Mechanism of QCS and its ingredients in the repair of the intestinal mucosal barrier and in the improvement of the damaged intestinal barrier, the expression of TJs, epithelial cell survival and migration, mucosal blood vessels, intestinal microbiota, and mucosal inflammation and immunity.

Lu *et al.* reported that FD4 in the colon of the colitis rats were increased as six times as that of normal rats and QCS could effectively decrease FD4 in the colon and promote ulcer healing of the colitis rats (Lu and Xie, 2010). Previous studies showed that the levels of DAO in serum were increased in the intestinal ischemia/reperfusion (IIR) rats, suggesting IIR could damage the mucosal integrity (Peng et al., 2018). GRb1 has antioxidant and antiapoptotic effects (Zheng et al., 2017). Chen et al. occluded the superior mesenteric artery for 75 min and re-perfused for 3 h to establish an IIR-intestinal epithelial injury model. Compared with the model group, GRb1 treatment group and pretreatment group could decrease the serum DAO and the expression of Akt and p-Akt. The protection of GRb1 could be eliminated by PI3K inhibitor, suggesting that GRb1 could decrease the intestinal permeability via PI3K/Akt pathway (Chen et al., 2019) (Figure 1).

### 4.2 QCS repairs the damaged intestinal barrier by elevating the expression of TJs

The disruption of intestinal TJs play an important role in the pathogenesis of UC (Tan et al., 2019). The decreased expression of ZO-1 and occludin proteins in UC patients leads to increased intestinal permeability and abundant pro-inflammatory factors invading the lamina propria of the colon, which triggers an intestinal immune response (Kim et al., 2018). Zhou et al. found QCS could increase the expressions of ZO-1 and occludin which were inhibited in the colitis mice induced by DSS (Zhou G. et al., 2021). Cai et al. found that LPS could suppress the expression of IPEC-J2 cells while this suppression could be reversed when the cells were pretreated by GA, an ingredient of gallnut (Liu et al., 2019; Cai et al., 2022).

### 4.3 Active ingredients of QCS repair the damaged intestinal barrier by regulating the epithelial cell survival and migration

Zhang et al. found purslane extract could not only decrease p-ERK, p-eIF2 α, Beclin1, and LC3II protein expression in IL-10^−/−^ model mice. But also reduce the damage of IECs by decreasing endoplasmic reticulum stress through the pERK-eIF2α/Beclin1-LC3II pathway (Zhang et al., 2022). Ginsenosides were found to significantly enhance colonic mucosal repair and promote intestinal mucosal healing in a TNBS-induced rat colitis model by activating the ERK and Rho-dependent pathways (Toyokawa et al., 2019). Indigo could increase IL-10 and IL-22 expression in isolated LP monocytes from colitis rats induced by TNBS and DSS. But this effect was not present in AhR^−/−^ mice. Moreover, indigo and indirubin could increase IL-10-producing CD4^+^ T cells and IL-22-producing CD3^+−^RORγt cells instead of CD4^+^Foxp3^+^Treg cells (Dudakov et al., 2015; Kawai et al., 2017; Wang et al., 2017).

Makoto et al. carried out a multicenter randomized controlled trial to investigate the safety and efficacy of IN in UC patients. A total of 86 patients were enrolled and given IN 0.5, 1.0, and 2.0 g or placebo for 8 weeks. The rates of mucosal healing were 13.6% in the placebo group, 56.5% in the 0.5 g IN group, 60.0% in the 1.0 g IN group, and 47.6% in the 2.0 g IN group (*p* = 0.0278 compared with placebo) (Naganuma et al., 2018). The *post hoc* analysis of this trial also demonstrated that the MH rate of IN was significantly higher than that of the placebo in the patients with steroid-dependent disease (*p* = 0.009) (Naganuma et al., 2020).

### 4.4 QCS repairs the damaged intestinal barrier by regulating the mucosal blood vessels

Intestinal EP is determined by the epithelia as well as the mucosal micro-vascular endothelia. The pathological micro-vascular endothelia cannot supply sufficient oxygen to the intestinal epithelia, destroy vasular function and accelerate the infiltration of inflammatory cells (Thornton and Solomon, 2002). Increased mucosal VP and decreased and slow blood flow also could lead to epithelia hypoxia (Taylor and Colgan, 2007), which induces increased pro-inflammatory cytokines resulting in intestinal epithelial cell injury and dissolution of TJs between goblet cells (Rezaie et al., 2007). Therefore, VP elevation may be the initial episode, earlier than increased EP, in UC occurrence and recurrence (Tolstanova et al., 2012), and VEGF inhibition can decrease VP and treat UC (Tolstanova et al., 2009). Wang S. et al. (2018) intervened DSS-colitis mice with QCS and found that QCS could improve colonic hypoxia and reduce colonic VP via VEGF/HIF-1a signaling pathway to improve vascular endothelial barrier function.

### 4.5 QCS repairs the damaged intestinal barrier by regulating intestinal microbiota

Intestinal microbiota is a complex community (Franzosa et al., 2019). It is also a crucial factor in intestinal mucosal injury (Kostic et al., 2014). The importance of intestinal flora in the occurrence and recurrence of UC has drawn increasing attention from the global scientific community. Increasing evidence has proven that the alteration of intestinal flora is a common feature in UC patients and mice colitis (Wlodarska et al., 2015). It has been generally accepted that UC is related to lower diversity of bacteria (Machiels et al., 2014; Prosberg et al., 2016; Knoll et al., 2017; Lopez-Siles et al., 2017) and decreased abundance of bacteria (Yao et al., 2016; Ricciuto et al., 2020). The damaged intestinal mucosal defense allows a large number of bacteria and their metabolites to enter the blood circulation and triggers a systemic immune response, which plays an extremely important role in the pathogenesis of UC (Kato et al., 2014; Liu et al., 2016). Therefore, the maintenance of intestinal microbial homeostasis is important to prevent the occurrence and recurrence of UC. Wen et al. observed whether QCS could treat UC via regulating colonic microbiota. They divided SD rats into 4 groups: normal group, DSS-induced colitis model group(M), M1 group and M2 group. The rats in M1 group were treated with DSS and filtrate of faeces of colitis rats. The rats in M2 group were treated with DSS and QCS-treated filtrate of faeces of colitis rats. They found the DAI score and the expression of TLR4 and NF-κB of M2 group were significantly lower than those of M group and M1 group. Additionally, QCS were found to improve the proliferation of *B. bifidum* and *B. thetaiotaomicron*. These results suggested that QCS could promote colon mucosa repair by improving the growth of *Bifidobacterium bifidum* and *Bacteroides thetaiotaomicron* and inhibiting TLR4/NF-kB signaling pathway (Wen et al., 2021). Harmful bacteria like *Bacteroidetes* and *Proteobacteria* producing TNF-α, IL-6, and IL-8 were found to increase, while beneficial bacterium like *Firmicutes* secreting IL-10 decrease in DSS-induced colitis mice (Ma et al., 2018; Liang et al., 2019). 16S rDNA sequence analysis proved that IN could adjust the composition of intestinal flora in colitis mice, especially related to the anaerobic Gram-positive bacteria of Turicibacter and *Peptococcus* (Liang et al., 2019; Yang et al., 2021). IN treatment could decrease the percentage of the harmful bacteria in colitis mice and restore the microbiota composition, which proves that IN could repair the DSS-induced gut microbiota imbalance (Liang et al., 2019). Indigo might exert its protective effects by increasing butyrate of the microbiota (Sun et al., 2020). The interaction between indigo and portulaca oleracea polysaccharide could promote short-chain fatty acid (SCFA) generation and metabolism (Fu et al., 2022). Wang et al. divided metronidazole-disposed-C57BL/6 mice into control, DSS, and DSS + ginseng pretreatment (28 mg/d/kg) groups. They found that the levels of three fecal endogenous metabolites including lactate, linoleic acid and malic acid of ginseng pretreatment group were significantly lower than those of DSS group, suggesting ginseng might repair the damaged intestinal barrier by regulating the intestinal microbiota (Wang CZ. et al., 2018).

### 4.6 QCS repairs the damaged intestinal barrier function by regulating mucosal inflammation and immunity

A fundamental experiment demonstrated that the inhibition of JAK/STAT pathway could benefit the integrity of the intestinal mucosa barrier (Chu et al., 2018; Soendergaard et al., 2018). Tofacitinib can inhibit JAK and is used to treat UC (De Vries et al., 2019). Researchers stimulated murine peritoneal macrophages with lipopolysaccharide (LPS), and they found the inhibition of JAK/STAT could decrease the secretion of inflammatory factors like IL-1β and IL-18 (Montoya et al., 2018). QCS and its ingredients, including indigo, ginsenoside R1, and GA, could exert the same effect as the inhibitor of JAK/STAT to alleviate inflammation and protect the intestinal mucosal barrier (Yu et al., 2021). In addition, GA has shown its antibacterial, anti-inflammatory, and antioxidant activity *in vitro* (Phonsatta et al., 2017; Cai et al., 2022). IgA produced by B cells, is a strong immunoglobulin on goblet, and protect the intestinal mucosal barrier from microorganism invasion (Pabst, 2012). IgA synthesis and secretion is the most recognized characteristic in mucosal immunity (Lycke and Bemark, 2017). Ginsenoside relieves the immune disorder in three ways: in the spleen, it can reverse proinflammatory and anti-inflammatory lymphocyte subsets ratio; in the intestine, it can stimulate CD4^+^ T cells to produce mucosal beneficial cytokines; and on the surface of goblet, it can assist B cells to secrete IgA to help mucin expression and the expression of TJs (Zhou R. et al., 2021). The decreased secretion of mucin by goblet cells weakens the intestinal mucus barrier and further impairs the function of the intestinal barrier (Zheng et al., 2019). GA could ameliorate dimethylhydrazine (DMH)-induced colonic inflammation, mucin depletion and intestinal epithelial cells’ oxidative stress, proliferation, and apoptosis disintegration in Wistar rats by upregulating the glutathione levels and activities of detoxifying enzymes (Shree et al., 2020).

## 5 Summary and prospect

The studies of mucosal healing, have developed rapidly in the past 10 years. The new perspectives of mucosal healing include new diagnostic tools like endomicroscopy, new etiology learning of intestinal inflammation like autoimmune response, and new biomarkers to predict mucosal healing like molecular markers. Based on the studies of QCS and its active ingredients, the mechanisms of QCS on repairing the intestinal mucosal barrier include suppression of colonic permeability, up-regulation of TJs, epithelial cell survival and migration regulation, mucosal blood vessels regulation, intestinal microbiota regulation, and mucosal inflammation and immunity regulation. Some of the above mechanisms and clinical results were concluded from the studies on the active ingredients of QCS instead of QCS. Whether the effects of the active ingredients could reflect the effect of the whole formula has not been clear. Therefore, it is necessary to study the mechanisms and the efficacy of the whole formula in the future. Additionally, the available clinical results of QCS are all from short term clinical trials. The long-term clinical effectiveness and safety of QCS are unknown. It is essential to conduct long-term clinical trial to provide substantial evidence for treating UC with QCS.
